# Like Father, Like Son: Assessment of the Morphological Affinities of A.L. 288–1 (*A. afarensis*), Sts 7 (*A. africanus*) and Omo 119–73–2718 (*Australopithecus* sp.) through a Three-Dimensional Shape Analysis of the Shoulder Joint

**DOI:** 10.1371/journal.pone.0117408

**Published:** 2015-02-04

**Authors:** Julia Arias-Martorell, Josep Maria Potau, Gaëlle Bello-Hellegouarch, Alejandro Pérez-Pérez

**Affiliations:** 1 Departament de Biologia Animal, Universitat de Barcelona, Barcelona, Spain; 2 Departament d’Obstetrícia, Ginecologia, Pediatria, Radiologia i Anatomia, Universitat de Barcelona, Barcelona, Spain; Museo Nazionale Preistorico Etnografico ‘L. Pigorini’, ITALY

## Abstract

The postcranial evidence for the *Australopithecus* genus indicates that australopiths were able bipeds; however, the morphology of the forelimbs and particularly that of the shoulder girdle suggests that they were partially adapted to an arboreal lifestyle. The nature of such arboreal adaptations is still unclear, as are the kind of arboreal behaviors in which australopiths might have engaged. In this study we analyzed the shape of the shoulder joint (proximal humerus and glenoid cavity of the scapula) of three australopith specimens: A.L. 288–1 (*A. afarensis*), Sts 7 (*A. africanus*) and Omo 119–73–2718 (*Australopithecus* sp.) with three-dimensional geometric morphometrics. The morphology of the specimens was compared with that of a wide array of living anthropoid taxa and some additional fossil hominins (the *Homo erectus* specimen KNM-WT 15000 and the *H. neanderthalensis* specimen Tabun 1). Our results indicate that A.L. 288–1 shows mosaic traits resembling *H. sapiens* and *Pongo*, whereas the Sts 7 shoulder is most similar to the arboreal apes and does not present affinities with *H. sapiens*. Omo 119–73–2718 exhibits morphological affinities with the more arboreal and partially suspensory New World monkey *Lagothrix*. The shoulder of the australopith specimens thus shows a combination of primitive and derived traits (humeral globularity, enhancement of internal and external rotation of the joint), related to use of the arm in overhead positions. The genus *Homo* specimens show overall affinities with *H. sapiens* at the shoulder, indicating full correspondence of these hominin shoulders with the modern human morphotype.

## Introduction

The African hominin *Australopithecus* is characterized by being adapted to an orthograde, or upright, body plan exhibiting a dorsoventrally flattened funnel-shaped thorax, as shown by cranial and trunk features (e.g. [1–5]). Pelvic and hindlimb evidence (well known at least for three australopith species: *Australopithecus afarensis*, *A*. *africanus* and *A*. *sediba* [6–8]) suggest that habitual bipedalism was common, with australopiths being largely obligate bipeds when on the ground [9–13]. However, features of the forelimb suggest that they also engaged in ape-like arboreal locomotor behaviors [4], [14–19]. As such, australopith specimens show high intermembral and brachial indices and relatively long and curved manual phalanges, which are usually related to arboreality (e.g. [16,20–25]). However, there is no consensus on the arboreal positional behavior of australopiths [4,14–16,24,25], and debate remains on what arboreal positional repertoire australopiths could have displayed (e.g., climbing behaviors, suspensory behaviors); some literature even stresses that these early hominins might not have engaged in arboreal behaviors at all [11–13,26–30].

The shoulder joint provides key anatomical information for making inferences on positional behaviors in living and fossil primates [31–36]. The glenohumeral joint comprises the proximal humerus and the glenoid cavity of the scapula. In the proximal humerus, two structures determine functionality: the humeral head, or articular surface of the humerus, and the major and minor tubercles, which bear the insertion sites of the rotator cuff muscles—subscapularis, supraspinatus, infraspinatus and teres minor—that control the movement and stability of the joint [37–39]. Primates displaying below-branch locomotor behaviors typically show large, protruding globular humeral articular surfaces, with relatively small tubercles lying well below the most superior aspect of the humeral head, which increases the mobility and the motion range of the glenohumeral joint [35,37,39–41]; *contra* [42,43]. Among suspensory apes and *Ateles*, distinctive proximal humeral morphologies can be discriminated in relation to the use of suspension. This group of primates also presents an ovate shape of the glenoid outline, with a smooth and moderately curved articular surface, possibly reflecting an adaptation to rapid limb motion with a high acceleration increment and a wide range of rotational shoulder movements [33,44]. Furthermore, arboreal quadrupedal primates have distinctive shoulder joint morphology compared with terrestrial quadrupeds, in that the shoulder joint is fairly globular (although not as much as in apes and *Ateles*), particularly in its medial aspect [40,41,45–47]. Humeral torsion has been linked to the dorsal positioning of the scapula on an orthograde thorax (wider mediolaterally and flattened anteroposteriorly), which causes the glenoid cavity of the scapula to face laterally. Concomitantly, the proximal humerus faces medially to maintain glenohumeral articulation. Some authors have suggested that the extensiveness of the humeral head is caused by the lateral migration of the lesser tubercle [40], whereas others maintain that surface extensiveness (i.e., mobility) and humeral torsion are independent features that may or may not appear together [41]. The shoulder joint is an essential part of the locomotor apparatus of primates in general, with well-established morphofunctional correlates, and thus serves as a good proxy to make functional inferences of the shoulder joint and locomotor behavior of fossil hominins.

Here we investigate the morphometric affinities of the glenohumeral joint of three australopith specimens from three different taxa (A.L. 288–1 [*A*. *afarensis*], Sts 7 [*A*. *africanus*], Omo 119–73–2718 [*Australopithecus* sp.]) to make locomotor inferences from a morphofunctional viewpoint. To do so, we use three-dimensional (3D) geometric morphometrics techniques to conduct quantitative analyses of the shape of the proximal humerus and the glenoid cavity of the scapula. Besides comparing the australopiths mentioned above with a varied array of extant anthropoids, we also compared their proximal humeri with those of other fossil hominoids and hominins to shed new light on the positional repertoire of these three australopith specimens.

## Material and Methods

The fossil sample includes five Plio-Pleistocene specimens (Table 1): the left proximal humerus (A.L. 288–1r) and the right glenoid cavity (A.L. 288–1l) of the specimen A.L. 288–1 (*A*. *afarensis*, Hadar, Kenya), the right proximal humerus and glenoid of Sts 7 (*A*. *africanus*, Sterkfontein, S. Africa), the left proximal humerus of Omo 119–73–2718 (*Australopithecus* sp., Omo, Kenya), the right humerus of Tabun 1 (*H*. *neanderthalensis*, Mount Carmel, Israel) and the right glenoid cavity of the scapula of KNM-WT 15000 (*H*. *erectus*, Nariokotome, Kenya). The scans of the fossil specimens were obtained from high-quality casts housed at the Center for the Study of Human Origins (CSHO) at the Anthropology Department of the New York University (NYU) and in Eric Delson’s collection at the American Museum of Natural History (AMNH, New York).

**Table 1:** Details of the fossil sample.

Taxon	Museum reference	Anatomical element^a^	Side	Period	Site	Museum(s)^b^
*Australopithecus afarensis* ^c^	AL 288–1r,l	PH/G	Left	Plio-Pleistocene	Hadar, Kenya	CSHO
*Australopithecus africanus* ^d^	Sts 7	PH/G	Right	Plio-Pleistocene	Sterkfontein, South Africa	CSHO
*Australopithecus* sp.^e^	Omo 119–73–2718	PH	Left	Plio-Pleistocene	Omo, Kenya	AMNHED
*Homo neanderthalensis* ^f^	Tabun 1	PH	Right	Plio-Pleistocene	Mount Carmel, Israel	CSHO
*Homo erectus* ^g^	KNM-WT 15000	G	Right	Plio-Pleistocene	Nariokotome, Kenya	CSHO

^a^PH, proximal humerus; G, glenoid cavity of the scapula.

^b^CSHO, Center for the Study of Human Origins, Anthropology Department, NYU (USA); AMNHED, Eric Delson’s collection at the American Museum of Natural History (AMNH, New York).

^c^[6,103], also known as “Lucy”.

^d^[7,94,95].

^e^Described as *A*. cf. *africanus* by Howell and Coppens [104], Howell [105], and McHenry and Temerin [106]; McHenry [107] later changed its attribution to *Homo* sp., but it was re-assigned to *Australopithecus* sp. by Larson [14].

^f^[108–111].

^g^[112,113].

The extant comparative sample for the proximal humerus included 133 individuals from eight primate taxa (Table 2): two New World monkeys, *Lagothrix* and *Ateles*, and five hominoids, hylobatids (including *Hylobates*, *Hoolock* and *Symphalangus*), *Pongo*, *Gorilla*, *Pan* and modern *H*. *sapiens* (including white American, black American and Khoisan individuals).

**Table 2:** Details of the comparative sample, including sample sizes (Total N), number of specimens per sex, as well as museum provenance.

	Proximal humerus				Glenoid cavity				
Taxon	Total N	M	F	n/a	Total N	M	F	n/a	Museum(s)^a^
Hoolock hoolock	**7**	1	4	2	**2**	1	-	1	AMNH
Nomascus concolor	**3**	2	1	-	**2**	1	1	-	AMNH
Hylobates agilis	**4**	1	3	-	**2**	-	2	-	AMNH
Hylobates moloch	**1**	0	1	-	**-**	-	-	-	AMNH
Symphalangus syndactylus	**4**	2	2	-	**4**	2	2	-	AMNH
*Hylobates* sp.	**1**	-	-	1	**1**	-	-	1	AMNH
Pongo pygmaeus	**18**	8	10	-	**14**	7	7	-	AMNH, UZH, PC
Pongo abelii	**2**	0	2	-	**2**	-	2	-	UZH
Pan troglodytes troglodytes	**9**	6	3	-	**6**	4	2	-	AMNH, PC
Pan troglodytes schweinfurtii	**8**	7	1	-	**8**	7	1		AMNH, PC
Gorilla gorilla	**15**	10	5	-	**14**	8	6	-	AMNH, PC
Ateles belzebuth	**1**	1	-	-	**1**	1	-	-	AMNH, UZH
Ateles geoffroyi	**6**	2	3	1	**5**	2	2	1	AMNH, UZH
Ateles paniscus	**1**	-	1	-	**-**	-	-	-	AMNH, UZH
Ateles fusciceps	**1**	-	1	-	**-**	-	-	-	AMNH, UZH
Lagothrix lagothrica	**15**	6	8	1	**11**	5	5	1	AMNH, UZH
*Lagothrix* sp.	**3**	1	-	2	**5**	2	1	2	AMNH, UZH
African American	12	6	6	-	**-**	-	-	-	AMNH
White American	**17**	12	5	-	**-**	-	-	-	AMNH
Bushmen	**5**	n/a	n/a	n/a	**-**	-	-	-	AMNH
White European	**-**	-	-	-	**19**	8	11	-	HCUB
Total	**133**				**96**				

^a^AMNH, American Museum of Natural History (NY, USA); UZH, Anthropologisches Institut und Museum of the Universität Zürich (Zurich, Switzerland); PC, Powell-Cotton Museum (Birchington, UK); HCUB, Hospital Clínic-Universitat de Barcelona; n/a, not available.

The extant comparative sample for the glenoid cavity included 96 individuals from the same primate taxa, which were mainly the associated glenoids to the former humeral specimens, with the exception of the *H*. *sapiens* sample, which included only white modern humans for the glenoid (Table 2).

All specimens were scanned at the American Museum of Natural History (AMNH, New York, USA), the Anthropologisches Institut und Museum of the Universität Zürich (UZH, Zurich, Switzerland) and the Powell-Cotton Museum (Birchington, UK). The modern European white sample of *H*. *sapiens* was provided by the Body Donation Service of the Universitat de Barcelona. Only wild-shot adult nonhuman primates were included in the sample, based on museum records, full epiphyseal fusion of the long bones and/or the emergence of the third molars. Right humeri were selected, except when missing or damaged, in which case left humeri were scanned and mirror-imaged during the editing process (for both extant primates and fossils).

### 3D geometric morphometrics

The bones (humeri and glenoids) were scanned with a 3D Next Engine laser surface scanner model 2020i, at a resolution of 0.1 mm space-point separation with a density of 40k (2x) points. The resulting triangular meshes were edited, and the models were then imported into the Landmark Editor software (v. 3.0.0.6) [48], and the landmark points were collected.

**Fig 1:**
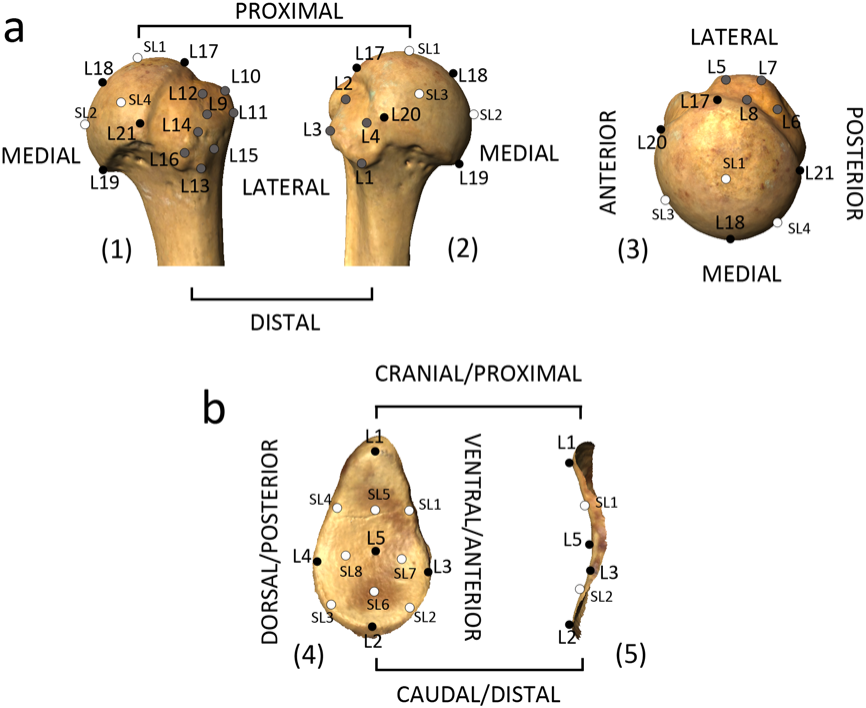
Landmark configurations used in the 3D geometric morphometric analysis of the proximal humerus and glenoid cavity of the scapula. a) configuration for the proximal humerus, in superior (1), posterior (2) and anterior (3) views; b) configuration for the glenoid cavity in frontal (4) and side (5) views. Black circles represent the homologous landmarks from the humeral head and the glenoid articular surface, gray circles the homologous landmarks from the tubercles of the proximal humerus, and white circles the semilandmarks (located only on the articular surface of the humerus and the glenoid).

We applied a protocol of 21 landmarks and four semilandmarks in the proximal humerus. This protocol, which is based on our previous studies [37,39], recorded the shape of the proximal articular surface as well as the shape of its greater and lesser tubercles (Fig. 1a; Table 2). Landmarks L1 to L16 corresponded to the tubercles: L1 to L4 outlined the subscapularis insertion site in the minor tubercle; L5 to L8 outlined the supraspinatus insertion on the major tubercle; L9 to L12 outlined the infraspinatus insertion; and L13 to L16 outlined the teres minor insertion. L17 to L21 served as the humeral head landmarks, which were recorded as three-point curves with Landmark Editor. This way, the four semilandmarks located on the articular surface were automatically equally spaced from the landmarks (L17 to L21) on the curves (Fig. 1a; Table 3).

**Table 3:** Landmark configurations for the proximal humerus and the glenoid cavity, indicating type^a^ of landmark and precise landmark description.

Landmark	Type	Description
PROXIMAL HUMERUS		
Tubercles		
L1	II	Distal end of the subscapularis insertion aspect
L2	II	Proximal end of the subscapularis insertion aspect
L3	II	Lateral point of the subscapularis insertion aspect
L4	II	Medial point of subscapularis insertion aspect
L5	II	Anterior end of the supraspinatus insertion aspect
L6	II	Posterior end the supraspinatus insertion aspect
L7	II	Lateral point the supraspinatus insertion aspect
L8	II	Medial point the supraspinatus insertion aspect
L9	II	Distal end of the infraspinatus insertion aspect
L10	II	Proximal end of the infraspinatus insertion aspect
L11	II	Lateral point of the infraspinatus insertion aspect
L12	II	Medial point of infraspinatus insertion aspect
L13	II	Distal end of the teres minor insertion aspect
L14	II	Proximal end of the teres minor insertion aspect
L15	II	Lateral point of the teres minor insertion aspect
L16	II	Medial point of teres minor insertion aspect
Articular surface		
L17	II	Intersection point between articular perimeter and the major tubercle prominence in a posterior/anterior view
L18	II	Maximum curvature point of the articular perimeter in the mediolateral and anteroposterior plane
L19	II	Most medial point of the articular perimeter
L20	II	Intersection point in the articular perimeter between the minor tubercle and the articular surface in superior view
L21	II	Intersection in the articular perimeter between the major tubercle and the articular surface in superior view
SL1	SL	Middle point between L17 and L18 on the articular surface
SL2	SL	Middle point between L18 and 19 on the articular surface
SL3	SL	Middle point between L20 and L18 on the articular surface
SL4	SL	Middle point between L21 and L18 on the articular surface
GLENOID CAVITY		
L1	II	Maximum curvature point on the proximal aspect
L2	II	Maximum curvature point on the distal aspect
L3	II	Maximum curvature the point on the anterior aspect
L4	II	Maximum curvature point on the posterior aspect
L5	II	Maximum craniocaudal curvature point in the center of the articular surface
SL1	SL	Middle point between L1 and L3
SL2	SL	Middle point between L3 and L2
SL3	SL	Middle point between L2 and L4
SL4	SL	Middle point between L4 and L1
SL5	SL	Middle point between L1 and L5
SL6	SL	Middle point between L2 and L5
SL7	SL	Middle point between L3 and L5
SL8	SL	Middle point between L4 and L5

^a^Landmark type (I, II and III) assignation according to Bookstein [49], O’Higgins [114] and Gunz et al. [50]; SL, semilandmark.

The protocol for the glenoid cavity was devised to represent its overall morphology (Fig. 1b; Table 3), with a total of five landmarks on the margin of the glenoid surface area, corresponding to the following points: maximum curvature on the proximal aspect, maximum curvature on the distal aspect, maximum curvature on the anterior aspect, maximum curvature on the posterior aspect, and maximum craniocaudal curvature in the center of the articular surface (Fig. 1b; Table 3). Four semilandmarks were collected on the margin (outline) of the articular surface of the glenoid, between the pairs of landmarks located in the surface outline (L1–L3, L2–L3, L1–L4, L2–L4), and four additional semilandmarks were recorded in the surface area, between L1 and L5, L2–L5, L3–L5 and L4–L5, to record the craniocaudal and anteroposterior curvatures of the surface (Fig. 1b; Table 3).

The landmark protocol was designed to meet the requirements of the fossil remains. Only external points of the insertion facets were recorded to avoid any erosion-related effects, which were only reported in some of the central parts of the supraspinatus and infraspinatus/teres minor facets. The landmarks on the humeral head captured the perimeters only where the surface was intact (preserving homology), and the surface of all specimens was preserved well enough to allow using automatically generated semilandmarks. To correct for the arbitrary placing of the latter, a sliding procedure was applied. In the humeral head, L17 to L21 served as anchors for sliding the semilandmarks (SL1–SL4), using the approach of minimizing the Procrustes distance. During the sliding process, each landmark was slid separately along tangent lines to the respective curve, removing the effect of arbitrary placement by minimizing the position of the semilandmarks with respect to the average shape of the sample [48–51]. Semilandmark sliding was performed with the Geomorph package (v. 1.1–4) for geometric morphometric analyses [52] developed for R (v. 3.0.2) [53].

### Multivariate analyses

Every analysis was applied to both subsets separately (proximal humerus and glenoid cavity): first, a generalized Procrustes analysis (GPA) was applied to the configurations of landmarks using the R software (S1). The GPA registered the raw coordinates of the landmarks with respect to one another by rotating, scaling and translating their configurations to minimize the sum of square differences among them [54,55] and then projected them onto the tangent space. Afterwards, a between-group Principal Components Analysis (bgPCA) was conducted in MorphoJ (v. 1.06a) [56] to explore major patterns of shape variation among taxa [57]; the scores for the fossil specimens were computed manually. MorphoJ computed a PCA on the covariance matrix of the group average shapes, and the resulting PC coefficients were then used with the dataset of the individual observations to plot the scatter of the specimens. Shape changes were explored by plotting the first principal component (PC1) against the second one (PC2) derived by the bgPCA (see also below). A minimum spanning tree (MST) based on Procrustes distances (calculated as the square root of the sum of square difference between two landmark configurations [58]) was applied to the bgPCA showing the closest morphological relationships between group centroids. Finally, a dendogram derived from a hierarchical cluster analysis (based on Ward’s method and conducted in PAST v.3, [59]) using Procrustes coordinates of group centroids (i.e., the whole shape) was used to explore the closest morphometric similarities between the extant groups and the fossil specimens in the morphospace.

Allometric multivariate regressions of centroid size (CS) against Procrustes coordinates were computed for each dataset. The Procrustes coordinates account for the whole shape in the morphospace, and the regression model computes a vector of regression scores for each independent variable (CS in this case) for all sample observations. Then, the vectors can be interpreted as shape variables with the strongest associations with the independent variable [60]. This process thus yields a holistic exploration of the relationship between size and shape [58]. A size-shape PCA (including the fossil taxa in the analyses) was then computed for each regression (proximal humerus and glenoid cavity) using the residuals of the shape-CS regression to explore the position of the fossil individuals with respect to their expected modern shape [61].

A multivariate regression between torsion angle and proximal humeral shape (as Procrustes coordinates) was conducted to explore the relationship between these two variables. A boxplot was used to illustrate the values of torsion in each extant group and the fossils, indicating the mean and the dispersion ranges for the extant taxa. Torsion angles were calculated in the virtual models following Larson [62,63]. Humeral torsion is the orientation of the humeral heads (measured as a line dividing it in two halves) relative to the mediolateral axis of the distal humerus. Ninety degrees instead of 0° was measured in humeral heads facing posteriorly (e.g., in *Lagothrix*) to enable direct comparison with previous works on humeral torsion [62,63]. As such, torsion values for the fossils were extracted from Larson [62] and incorporated into our data. Only torsion values for A.L. 288–1r (*A*. *afarensis*), Sts 7 (*A*. *africanus*) and Omo 119–73–2718 (*Australopithecus* sp.) were available.

For visualization, the extreme shapes of the first two axes were extracted and explored in the bgPCA. To explore the particular aspects of shape related to the factors tested, shapes at the end of the horizontal axis (independent factor) were extracted in the regressions as well. A generic mesh model representative of the mean shape of each analysis (bgPCA, CS regression and torsion regression) provided by MorphoJ was constructed in Landmark Editor, and the extreme shapes were then warped to it using Landmark Editor.

## Results

### Proximal humerus

**Fig 2:**
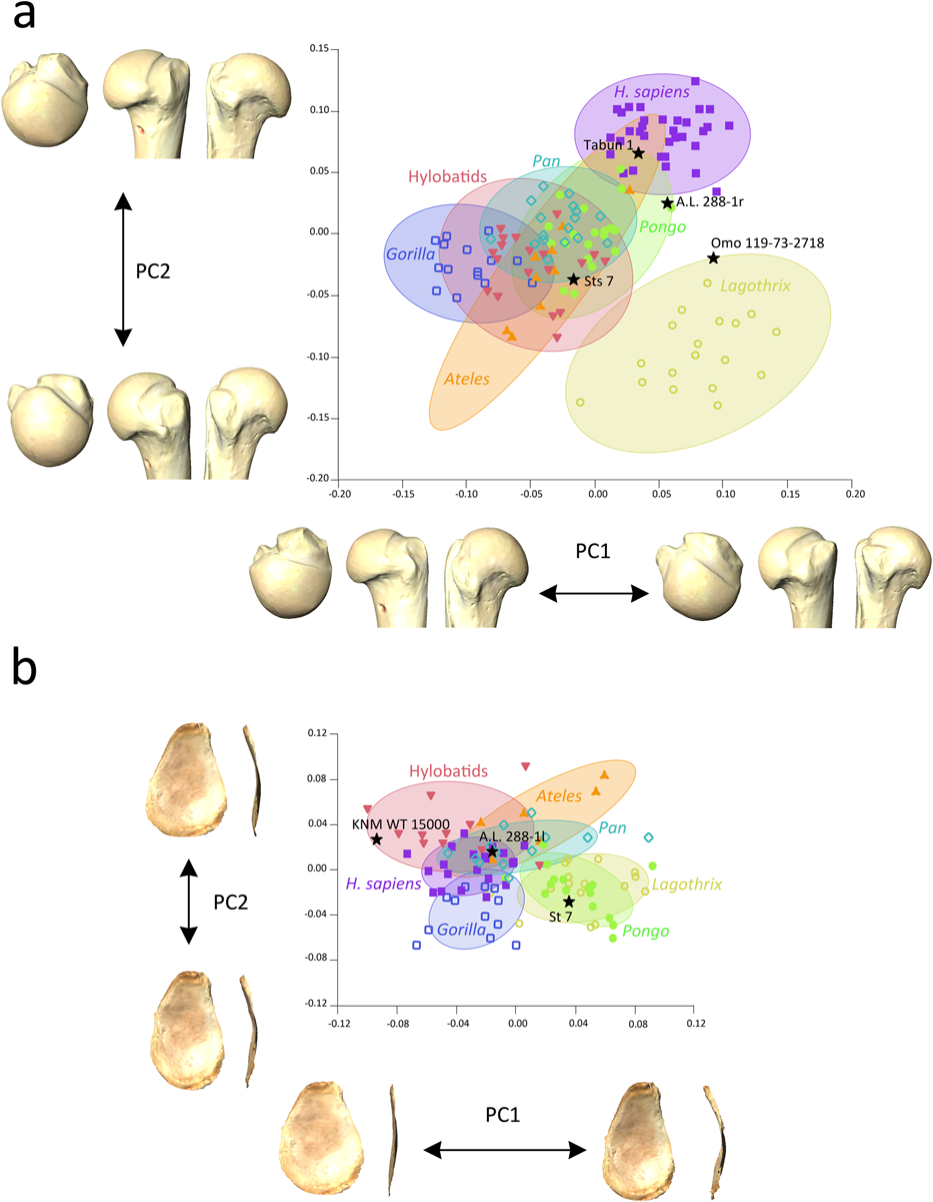
Results of the between-group Principal Components Analyses (bgPCA) depicted as a bivariate plot of the two first bgPCA scores for each individual (bgPC2 vs. bgPC1). a) proximal humeral shape: humeral head shape changes along each axis are shown in posterior, anterior and superior views at their extreme ends; b) glenoid cavity shape: glenoid shape changes along each axis are shown in frontal and side views at their extreme ends. 95% equal frequency ellipses of the groups are depicted.

The bgPCA for the proximal humerus yielded six principal components (PCs) explaining 100% of the variance (Table 4). For PC1 (explaining 33.45% of the variance) *Lagothrix* falls on the positive end of the axis, as does the group of modern humans, with the two groups greatly overlapping. At the negative end, the group of apes clusters together with *Ateles* (Fig. 2a). Vectors of shape change towards the positive end of the PC1 are driven by the presence of large tubercles with respect to the articular surface. The bicipital groove is wide, related to a relatively anteriorly positioned minor tubercle, which appears rounded and large overall. The articular surface does not show progression onto the intertubercular space, but exhibits a lateral expansion towards it, conferring an oval outline to the articular surface. Nevertheless, its shape is overall rounded and fairly globular. In contrast, the shape changes towards the negative end of the axis represented by hominoids, and *Ateles* show a more globular (on the superior aspect) and enlarged articular surface with respect to the tubercles. The minor tubercle is laterally positioned, which in turn affects the bicipital groove, which becomes deep and narrow. The minor tubercle is also smaller and spindle-shaped, and the supraspinatus insertion site appears reduced in the major tubercle.

**Table 4:** PCs variance, total variance for each PC and cumulative variance.

	Variance	% Total variance	% Cumulative
Proximal humerus			
PC1	0.00392776	33.45	33.45
PC2	0.00372287	31.71	65.16
PC3	0.00193589	16.49	81.64
C4	0.00096323	8.20	89.85
PC5	0.00066852	5.69	95.54
PC6	0.00052354	4.46	100
Glenoid cavity			
PC1	0.00182942	42.26	42.26
PC2	0.00110351	25.49	67.76
PC3	0.00042648	9.85	77.61
PC4	0.00041352	9.55	87.16
PC5	0.00030056	6.94	94.11
PC6	0.00025498	5.89	100

For PC2 (31.71% of the variance) the *H*. *sapiens* group exhibits the most positive values, although some overlap exists with the *Pongo* and *Ateles* groups. The latter group overlaps with all of the hominoid taxa due to its wide dispersion range. Some *Lagothrix* specimens exhibit the most negative values for PC2. The vectors of shape change towards the positive end of the axis and show a relative flattening of the articular surface on its proximal aspect, which displays a pronounced lateral expansion towards the bicipital groove, even though it appears narrow and deep. The overall aspect of the articular surface is globular and rounded, but it displays an oval outline. The major tubercle is reduced with little space for the supraspinatus insertion, and the infraspinatus insertion is oriented cranially with respect to the shape of the negative end of the PC. The teres minor insertion is more medially positioned and does not exhibit the laterally protruding tubercle seen in the other morphologies. The minor tubercle is overall smaller, tilted latero-medially, and exhibits an oblique orientation. Towards the negative end of the axis, the taxa show nearly spherical articular surface contours (in anterior and posterior views), being rounded and protruding medially and superiorly, as well as having a shorter medio-lateral diameter. The tubercles are laterally oriented, and the bicipital groove is slightly less deep and narrow than previously described. The insertion site for the supraspinatus is large and triangular, and the infraspinatus insertion is not oriented cranially, but faces posteriorly. The minor tubercle is spindle-shaped and its major axis displays a primarily proximo-distal orientation.

The fossil hominins fall mostly within the ellipses of the orthograde taxa (Fig. 2a): A.L. 288–1r (*A*. *afarensis*) falls within the ellipse of *Pongo*, near the modern human variation and overlapping with one *Pongo* specimen; Tabun 1 (*H*. *neanderthalensis*) falls within the overlapping zone of *Pongo*, *H*. *sapiens* and *Ateles*; Sts 7 (*A*. *africanus*) is situated in the middle of the orthograde main scatter of points, in the overlapping ellipses of *Pongo*, *Pan*, hylobatids and *Ateles*. However, Omo 119–73–2718 (*Australopithecus* sp.) falls at the edge of the 95% equal frequency ellipse of *Lagothrix*, although it overlaps with the group of modern humans for PC1.

**Fig 3:**
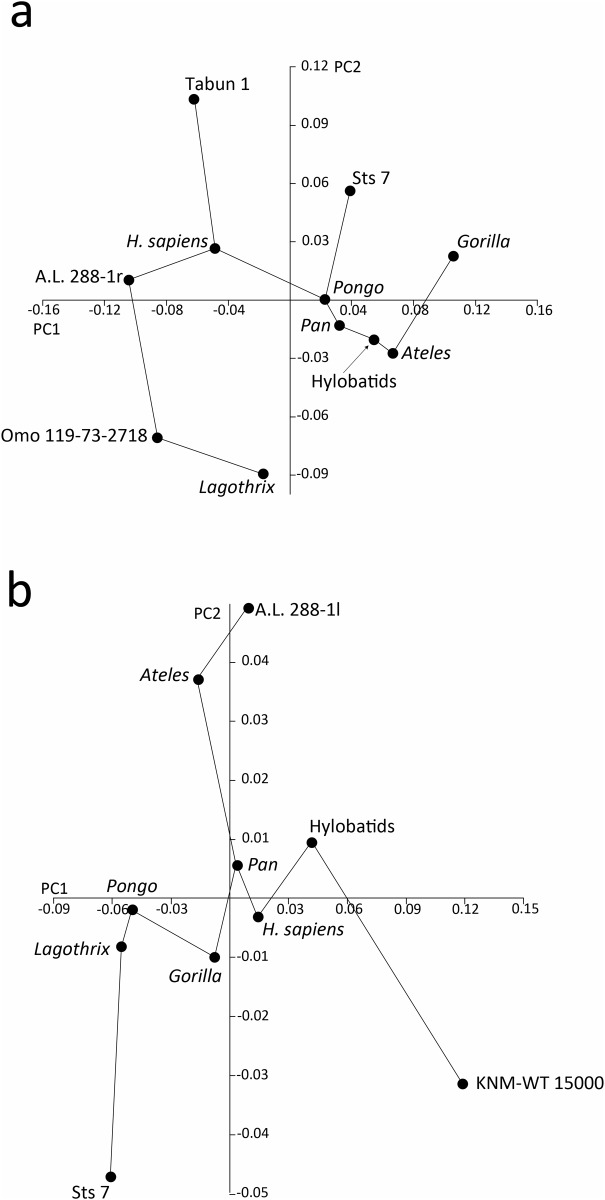
Results of the between-group Principal Components Analyses, depicted as a minimum spanning tree (bgPCA-MST). Scores for the extant taxa centroids and the scores for the fossil specimens are shown: a) bgPCA-MST of the proximal humerus, b) bgPCA-MST of the glenoid cavity.

In the MST-PCA (Fig. 3a) A.L. 288–1r more closely resembles modern humans in the PC1 vs. PC2 graph; it also exhibits the shortest Procrustes distance to this group when the overall shape is taken into account (Table 5a). Sts 7 (*A*. *africanus*) more closely resembles *Pongo*, also exhibiting the shortest Procrustes distance to it for the whole shape. Omo 119–73–2718 (*Australopithecus* sp.) appears between *Lagothrix* and the other hominin A.L. 288–1r, but it exhibits the shortest distance to the extant *Lagothrix*. Tabun 1 more closely resembles modern humans, exhibiting the shortest distance to this group as well.

**Table 5:** Matrix of Procrustes distances among pair of groups for A) the proximal humerus and B) the glenoid cavity shape, including extant taxon centroids and fossil specimens.

Proximal humerus										
Taxon/specimen	Tabun 1	Ateles	AL 288–1r	Sts 7	Omo 119–73–1827	Gorilla	H. sapiens	Hylobatids	Lagothrix	Pan
Ateles	0.2024									
AL 288–1r	0.1754	0.2098								
Sts 7	0.1675	0.1658	0.2177							
Omo 119–73–1827	0.1896	0.1887	0.1706	0.2097						
Gorilla	0.2052	0.1311	0.2339	0.1528	0.2286					
H. sapiens	**0.1319**	0.1598	**0.1671**	0.1932	0.1548	0.1917				
Hylobatids	0.1936	0.0836	0.1943	0.1778	0.1870	0.1308	0.1623			
Lagothrix	0.2138	0.1617	0.1982	0.1791	**0.1343**	0.1959	0.1813	0.1704		
Pan	0.1847	0.1279	0.1809	0.1725	0.1655	0.1019	0.1331	0.1254	0.1600	
Pongo	0.1540	0.0896	0.1773	**0.1492**	0.1616	0.131	0.117	0.1022	0.1445	0.1078

Glenoid cavity										
Taxon/specimen	Ateles	AL 288–1l	Sts 7	Gorilla	KNM-WT-15000	H. sapiens	Hylobatids	Lagothrix	Pan	
AL 288–1l	0.0762									
Sts 7	0.1039	0.1262								
Gorilla	0.0914	**0.0799**	0.0935							
KNM-WT 15000	0.1563	0.1422	0.1844	0.1419						
H. sapiens	0.075	0.0832	0.106	0.0535	0.1203					
Hylobatids	0.0718	0.0833	0.1248	0.0839	**0.0982**	0.0491				
Lagothrix	0.0748	0.1042	**0.0689**	0.0794	0.1777	0.0825	0.1082			
Pan	0.0559	0.082	0.1007	0.0753	0.1269	0.0555	0.0604	0.072		
Pongo	0.0755	0.0971	0.0769	0.0755	0.1745	0.0812	0.1059	0.0356	0.0695	

The shortest distances between fossils and extant taxa centroids are highlighted in bold numbers.

**Fig 4:**
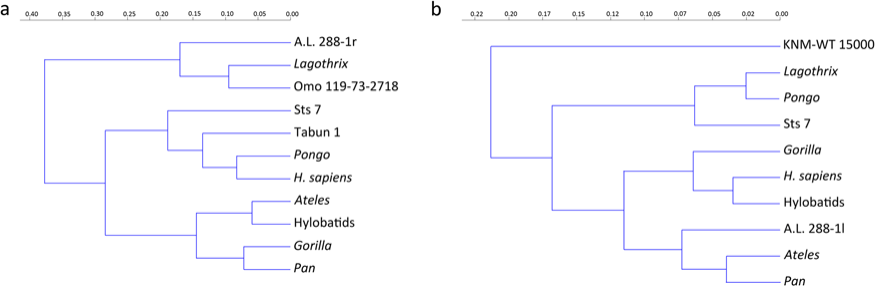
Results of the hierarchical cluster analysis (Ward’s method) based on the group centroids (extant taxa) and scores (fossil individuals) delivered by the bgPCA analyses. a) Cluster for the proximal humerus results; b) cluster for the glenoid cavity results.

The cluster analysis based on Procrustes coordinates (Fig. 4a, S1 Table) separates two major clusters, one grouping the *Lagothrix* with two hominins, A.L. 288–1r and Omo 119–73–2718, and the other encompassing the orthograde taxa and the remaining hominins. Within the latter, two subclusters are distinguished, one grouping the hylobatids, the African great apes and *Ateles*, and the other encompassing *Pongo* and *H*. *sapiens* with Sts 7 and Tabun 1.

**Fig 5:**
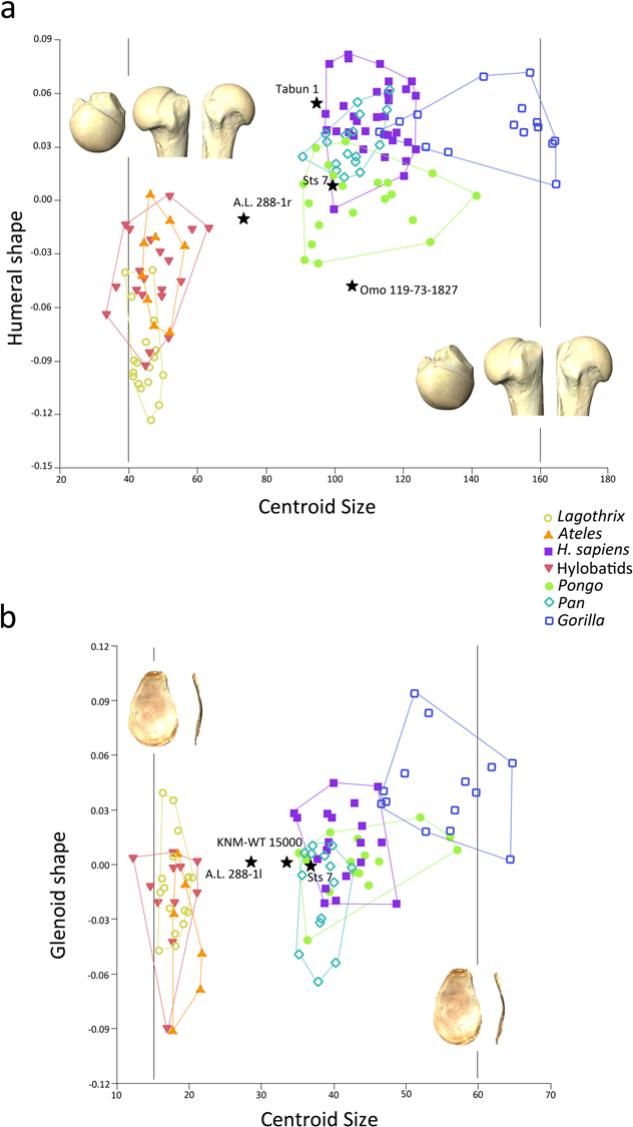
Bivariate plot of the results of the linear regression of centroid size (CS) onto a) proximal humeral shape and b) glenoid cavity shape. In a) the warps represent the shapes at a CS of 40 in the lower end of the regression slope, broadly corresponding to the smaller taxa (*Ateles*, *Lagothrix* and hylobatids) and at 160 (higher end of the regression slope), mainly corresponding to *Gorilla*; in b) the warps represent the shapes at a CS of 15 in the lower end of the regression slope, broadly corresponding to the smaller taxa (*Ateles*, *Lagothrix* and hylobatids) and at 60 (higher end of the regression slope), mainly corresponding to *Gorilla*. Convex hulls depict the range of dispersion of the different groups.

The regression of shape onto CS was significant at P < 0.000, explaining 9.43% of the variance. The graph shows completely different allometric trajectories between the smaller taxa and the larger ones. On the lower end of the regression slope (Fig. 5a), the taxa with lower values of CS (*Lagothrix*, *Ateles* and hylobatids) exhibited a proximal humeral shape with round and fairly globular articular surfaces, exhibiting a maximum expansion towards the bicipital groove, with relatively large tubercles with a somewhat wide bicipital groove. The insertions in the greater tubercle appeared proximodistally aligned, with a triangular shape of the supraspinatus insertion, a cranial orientation of the infraspinatus and a laterally placed and big teres minor insertion. On the higher end of the regression slope, taxa with high CS values (*Gorilla* exhibited the higher CS values) exhibited a medially shorter articular surface that does not protrude excessively above the tubercles. The laterally flaring and large greater tubercle exhibits a smaller supraspinatus insertion, a big infraspinatus insertion and a more medially positioned teres minor insertion. A.L. 288–1r falls between the two clusters of extant taxa, although positioned closer to the group of hylobatids, *Lagothrix* and *Ateles*. Sts 7 exhibits an expected proximal humeral shape for its CS value, falling well within the ranges of Pongo. Omo 119–73–2718, however, exhibits a much higher CS value (in the ranges of the great ape taxa) than expected for its proximal humeral shape (in the ranges of the smaller taxa: hylobatids, *Ateles* and *Lagothrix*).

**Fig 6:**
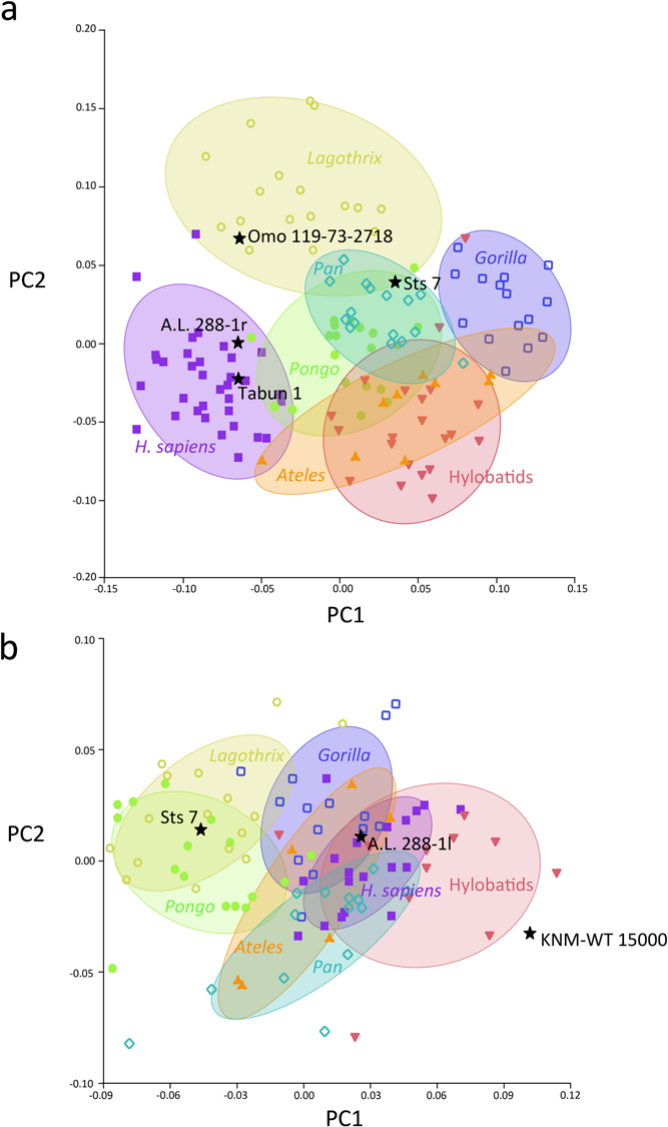
PC1 *vs*. PC2 scatterplots of size-shape PCAs computed with the regression residuals of CS against shape. a) Size-shape PCA of the proximal humeral shape. PC1 explains 27.95% of the variance and PC2, 16.93%; b) size-shape PCA of the glenoid cavity shape. PC1 explains 34.60% of the variance and PC2, 15.64%.

The size-shape PCA computed with the residuals of the previous regression (Fig. 6a) shows similar relationships between the fossils and the extant groups than those found for the bgPCA (Fig. 2a), except for the position of A.L. 288–1r, which clearly falls within the dispersion ranges of modern humans, together with Tabun 1. Sts 7 falls within the overlapping ellipses of *Pan* and *Pongo* and at the edge of *Lagothrix*, while Omo 119–73–2718 falls within the *Lagothrix* ellipse.

**Fig 7:**
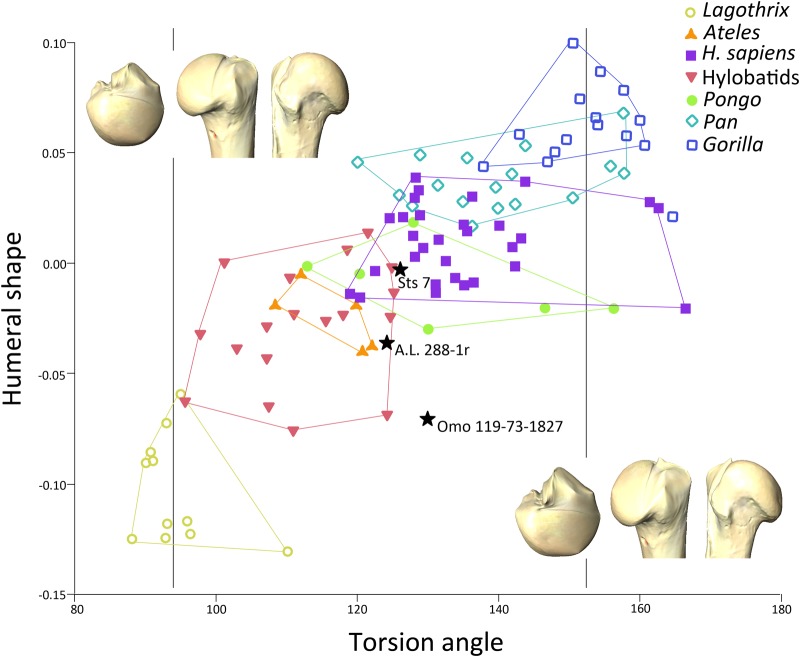
Bivariate plot of the results of the linear regression analysis of humeral torsion on proximal humeral shape. Convex hulls depict the range of dispersion of the different groups. Warps represent the mean torsion angle of Lagothrix (94.17) on the lower end of the slope and the mean torsion of *Gorilla* (152.71) in the higher end of the slope.

**Fig 8:**
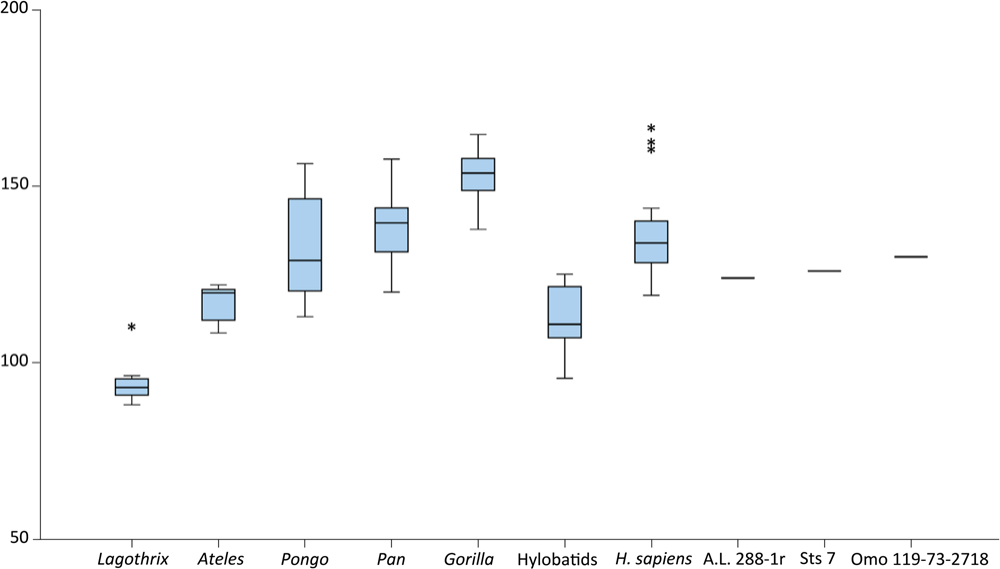
Boxplot of the means and dispersion ranges of humeral torsion values per living taxon, including the value for the fossil hominins.

Mean values of the genera for humeral torsion are reported in Table 6, with hylobatids displaying the lower values of humeral torsion for the extant hominoids and the African great apes (particularly *Gorilla*) and humans displaying the higher values, agreeing well with previous results [60,61]. The regression analysis between torsion and proximal humeral shape was significant (P < 0.001), and torsion explained 8.70% of variance. The regression graph (Fig. 7) shows the African apes and *H*. *sapiens* at the higher (positive) end of the slope, corresponding to higher values of torsion, and *Lagothrix* placed in the lower (negative) end of the slope, displaying virtually no torsion. A.L. 288–1r displays higher torsion values than expected for its shape, although it fits well within the hylobatids and *Ateles* ranges. Sts 7 position on the regression slope is within the ranges of the orthograde taxa, specifically for *Pongo*, but it is also on the lower end of the modern human ranges. However, Omo 119–73–2718 clearly exhibits higher torsion angles than expected for its proximal humeral shape, as evidenced by its lower position (within the higher ranges of *Lagothrix* but also the lower ranges of hylobatids) in the regression slope. The overall aspects of proximal humeral shape that are related to humeral torsion as it increases (i.e., the features that change as torsion values increase remain the same but are emphasized) are the lateral migration of the lesser tubercle, a lateral flaring of the greater tubercle, and a medially short (and even flat) articular surface that exhibits a great anteroposterior diameter (superior view) and does not protrude excessively above the tubercles. The boxplot (Fig. 8) showing torsion values per group (including the fossil specimens) illustrates that the dispersion ranges of the great apes and *Ateles* broadly overlapped, with *Pongo* being the taxon with greater dispersion ranges. Hylobatids and *Lagothrix* exhibited lower positions agreeing with their lower torsion values, with *Lagothrix* being the taxon with lesser dispersion ranges. The fossils fell within the dispersion ranges of the apes (with the exception of *Gorilla*) and *Ateles*, farther away from *Lagothrix*.

**Table 6:** Humeral torsion values per genus means with sample sizes (N) and standard deviations (SD).

Genus	Mean	N	SD
Ateles	116.63	5	6.01
Lagothrix	94.17	11	5.86
Cebus	95.77	17	2.16
Pongo	132.36	6	16.24
Pan	139.41	17	11.21
Gorilla	152.71	15	7.11
Hylobatids	112.40	18	9.63
H. sapiens	135.32	33	11.14
Total	124.51	122	21.74

### Glenoid cavity

The bgPCA for the glenoid cavity yielded six principal components explaining 100% of the variance (Table 4). For PC1 (explaining 42.27% of the variance) *Pongo* and *Lagothrix* (virtually occupying the same position on the plot) overlap with *Pan* and *Ateles* and fall towards the positive end of the axis, with the rest of the groups (*Gorilla*, *H*. *sapiens* and hylobatids) overlapping towards the negative end (Fig. 2b). Hylobatid specimens show the most negative values for PC1. In general, wide dispersion ranges exist for all taxa, producing a great overlap between group ellipses and individuals. The shape change vectors towards the positive end of PC1 show that taxa situated towards this end exhibit narrow and cranially elongated glenoid surfaces, with a relatively pronounced cranio-caudal curvature. In contrast, towards the negative end of the axis, the taxa exhibit nearly round glenoid surfaces with a great widening of the caudal aspect, also exhibiting great flatness and wide oval outline.

PC2 (explaining 25.49% of the variance) shows a wide overlap of taxa (Fig. 2b). As shown by the vectors of shape changes towards the positive end of the axis (Fig. 2b), the taxa at this end exhibit a slight curvature of the glenoid surface, with a piriform (pear-shaped) outline because of a widening of the caudal part. Towards the negative end, the taxa have more elongated glenoid surfaces, curved more cranio-caudally than at the positive end, with a narrow oval outline.

A.L. 288–1l (*A*. *afarensis*) falls within the main scatter of points of the orthograde taxa, in the overlapping ellipses of *H*. *sapiens*, *Pan*, *Ateles* and hylobatids; Sts 7 (*A*. *africanus*) falls in the ellipses of both *Pongo* and *Lagothrix*; and KNM-ER 15000 (*H*. *erectus*) is situated among the hylobatids, showing very negative values for PC1 (Fig. 2b). In the MST-PCA (Fig. 3b) A.L. 288–1l appears more similar to *Ateles*, Sts 7 to *Lagothrix* and KNM-WT 15000 (despite being the most distantly situated taxon) to the hylobatids. Procrustes distances among groups (Table 5b) indicate that A.L. 288–1l exhibits the shortest distance to *Gorilla*, but is nearly the same distance from groups of *Pan*, H. *sapiens* and the hylobatids. Sts 7 is instead more closely situated to *Lagothrix* than to any other taxon, and KNM-WT 15000 exhibits the shortest distance to the hylobatids.

The cluster analysis based on Procrustes coordinates (S2 Table) separates KNM-WT from the rest of the taxa and positions it as an outgroup (Fig. 4b). Within the major cluster, two subclusters are present: one groups Sts 7 with *Lagothrix* and *Pongo*, and the other is again subdivided into two subclusters, one grouping A.L. 288–1l with *Ateles* and *Pan* and the other assembling extant taxa (*Gorilla*, hylobatids and *H*. *sapiens*).

The regression of shape onto CS was significant at P < 0.05, explaining 4.84% of the variance. The shape changes along the regression slope are slight, since very little of the variance is explained. On the lower end of the regression slope the taxa with low values of CS (*Lagothrix*, *Ateles* and hylobatids) exhibit a glenoid cavity shape with an oval outline, with a slight notch present on the anterior aspect and a relatively pronounced cranio-caudal curvature. Towards the higher end of the regression slope the taxa exhibit glenoid surfaces with a narrower caudal portion and a more pronounced cranio-caudal curvature (Fig. 5b). A.L. 288–1l falls between the two clusters of extant taxa, although it is positioned closer to the group of great apes. Sts 7 exhibits an expected proximal humeral shape for its CS value, falling well within the ranges of *H*. *sapiens*, *Pan* and *Pongo*. KNM-WT 15000 exhibits a glenoid shape well within the ranges of the great apes (except *Gorilla*), but with a CS value in the lower end of the great ape distribution.

The size-shape PCA computed with the residuals of the previous regression (Fig. 6a) shows similar relationships between the fossils and the extant groups compared with those found for the bgPCA (Fig. 2b). KNM-WT 15000 falls near the range of variation of the hylobatids; A.L. 288–1l falls in the overlapping ellipses of *H*. *sapiens*, *Ateles*, hylobatids and *Gorilla*; and Sts 7 falls within the ellipses of *Lagothrix* and *Pongo*.

## Discussion

### The proximal humerus

**Fig 9:**
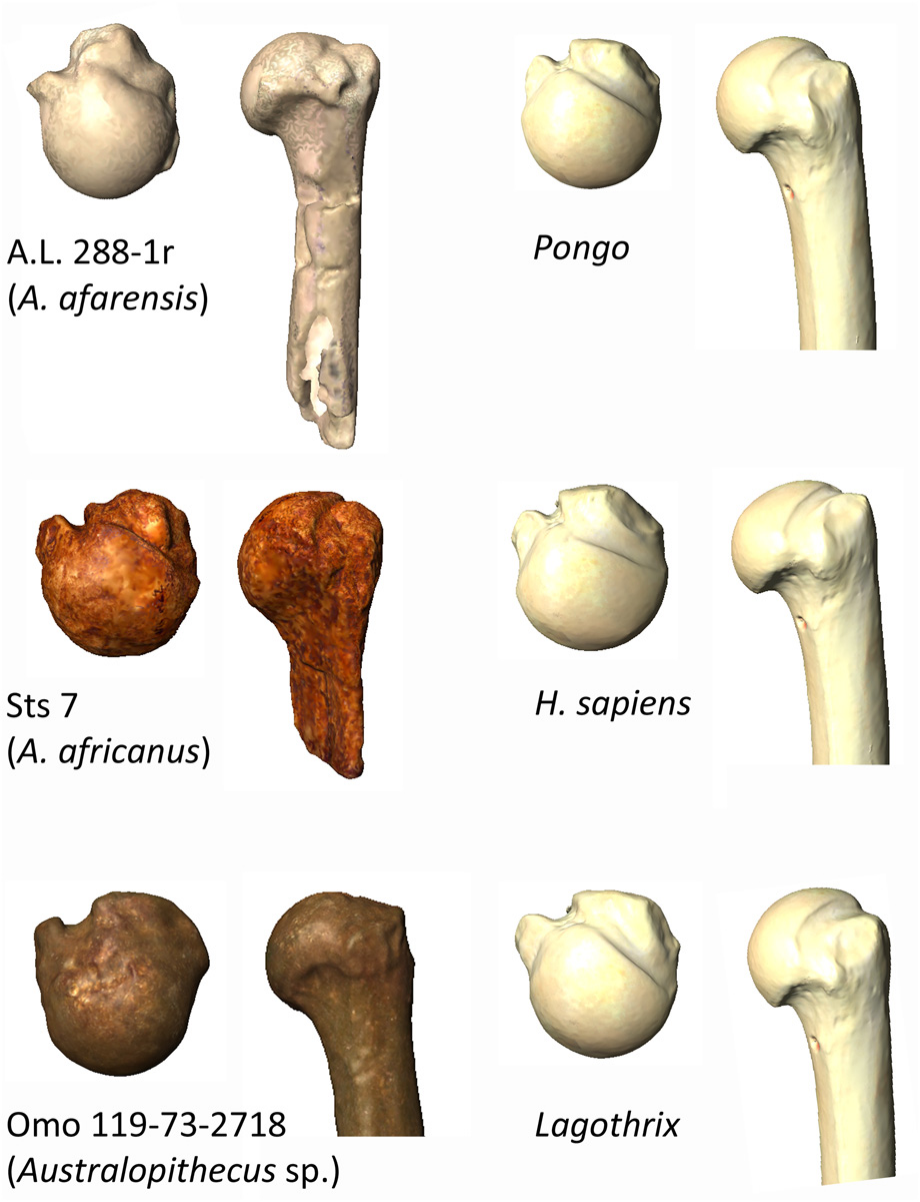
3D models of the three australopiths—A.L. 288–1r (*A*. *afarensis*), Sts 7 (*A*. *africanus*) and Omo 119–73–2718 (*Australopithecus* sp.)—proximal humeri included in the study with a sample of extant taxa. The humeri are shown in proximal and posterior views. *Pongo* is shown as a representative of the arboreal ape shape and *Lagothrix* as a representative of a more generalized arboreal shape. The humeri are at the same scale for interpretative purposes.

The proximal humerus morphology of A.L. 288–1r (*A*. *afarensis*) exhibits mixed characteristics, showing some affinities with the modern humans (Fig. 3a, Table 5) and *Pongo* in the bgPCA (Fig. 2a) and with the smaller taxa (hylobatids, *Ateles* and *Lagothrix*) (Figs. 4a, 5a and 7). With the arboreal apes and atelines, A.L. 288–1r shares the position and shape of the greater tubercle insertions, but the positioning of the humeral head with respect to the tubercles as well as its overall shape is more similar to the modern human morphotype (Fig. 9). A mosaic nature has been found for a number of postcranial structures in early hominins, including the forelimb and the shoulder region [14,64–66,67], and *A*. *afarensis* specimens have been described as showing more modern-looking characteristics than later australopiths for other cranial and postcranial regions (e.g., [68]). However, a study by Lague [61] on allometric changes in the distal humerus indicated that for particularly small early hominin specimens (such as A.L. 288–1r, to which the study specifically refers) if shape changes are analyzed without accounting for the size-shape variation of the comparison sample (i.e., modern humans), the morphological associations of this region could appear more human-like. If the regression of humeral shape onto CS (Fig. 5a) is considered, A.L. 288–1r exhibits a CS value more similar to the small taxa of the study and a proximal humeral shape within the upper ranges of those groups (*Ateles*, *Lagothrix*, hylobatids). The CS value is also well within the lower ranges of *Pongo*, but it is clearly far from the ranges of modern humans. However, in the size-shape PCA (the PCA of the residuals of the CS-shape regression, Fig. 6a), A.L. 288–1r does not differ morphologically from the modern humans for the two first PCs, appearing more human-like again than more modern (fossil) taxa (Sts7 and Omo 119–73–2718), as suggested by McHenry and Brown [68].

Sts 7 (*A*. *africanus*) is overall most similar to the large arboreal hominoids, particularly *Pongo* [69] (Figs. 2a, 3a, 4a, 5a, 6a and Table 5), and shares a glenohumeral morphology related to high mobility in the joint with this taxon [35,40] (but see [42,43] for a different view), including relatively globular articular surfaces in the central and proximal aspects (Figs. 2b and 8). Such a shape is related to an enhanced range of circumduction of the arm, enabling ball-and-socket contact with the glenoid in the central and superior aspects of the articular surface of the humerus, possibly providing greater stabilization of the joint when the arm is abducted [37,39,70]. Moreover, Sts 7 shows a relative lateral placement of the teres minor insertion, as seen in the arboreal apes (Fig. 8, *Pongo*), and particularly *Pongo*, which even exhibits a slightly protruding tubercle. This condition is related to an enhancement of the teres minor muscle role as an external rotator (Fig. 2a). Powerful external rotation of the glenohumeral joint has been linked to the functional demands of arm-swinging and hoisting capabilities in the living taxon [37,39,71–73]. *Pongo* and modern humans overlap in a number of analyses (Figs. 2a, 3a 4a), indicating a closer morphological relationship between humans and orangutans than for humans and African great apes related to a relative reduction of the supraspinatus insertion. Such a pattern has also been observed for the morphology of the scapula, for which *Pongo* and *Homo* exhibit a reduced supraspinous fossa, suggesting a higher reliance on the infraspinatus muscle role in suspensory behaviors in *Pongo* over the pure abductor supraspinatus and enhanced speed and precision in humans related to manipulatory purposes [74]. The overall morphology of the proximal humerus in Sts 7 is more similar to that of *Pongo* (Fig. 9), especially the shape of the articular surface, which is medio-laterally short and quite globular on its superior aspect as in *Pongo*, which is functionally related to arm-rising behaviors (e.g., reaching, hanging) in the living taxon [37,39]. When allometry is taken into account (Fig. 6a), Sts 7 is placed further from modern humans and is situated within the ranges of the great apes (particularly *Pongo* and *Pan*). Thus, in spite of the morphological overlap between humans and *Pongo* in other analyses, the Sts 7 proximal humerus presents more ape-like features when size is controlled for, which suggests that this specimen’s proximal humeral morphology could be related to the retention of arboreal capabilities in its glenohumeral joint [14–16].

Omo 119–73–2718 (*Australopithecus* sp.) showed general morphometric affinities with *Lagothrix* (Figs. 2a, 3a, 4a, 6a and Table 5). This specimen resembles *Lagothrix* in the oval outline of the articular perimeter and the presence of relatively large tubercles respect to the humeral head, with a wide and shallow bicipital groove (Fig. 9; *Lagothrix*). The morphological association of Omo 119–73–2718 and *Lagothrix* could be related to *Lagothrix* standing out as an example of an intermediate condition for the proximal humerus between strict arboreal quadrupeds and suspensory taxa [39,75]. *Lagothrix* exhibits derived morphological aspects in the proximal humerus such as a rounder and less flattened articular surface of the humeral head with an increased globularity compared with quadrupeds, particularly in its superior aspect, that closely resembles *Ateles* and *Pongo* (Figs. 2a, 3a and 9). Differences between apes/*Ateles* (because *Ateles* mainly shares the proximal humeral morphotype with *Pongo* [39,46]) and *Lagothrix* are related to the moderate use of below-branch locomotor behaviors of the latter taxon. *Lagothrix* is capable of engaging in demanding arm circumduction behaviors, such as brachiation [76], without showing extreme adaptations to such behaviors or an orthograde body plan. As such, intermediate and generalized arboreal morphologies might be more representative of the basal morphotype from which hominins evolved than the suspension-derived extant great apes [39,41,77,78]. Recent evidence from the relatively primitive limb morphology of *Ardipithecus ramidus* (Late Miocene, 4.4 Ma, [64–66]) also builds on the contention that the last common ancestor of chimpanzees and humans could have exhibited a more primitive condition than previously expected from the suspension-derived morphologies of the living apes. Thus, the ancestors of hominins might have exhibited generalized arboreal traits, making it plausible for the analyzed australopith specimens to show mixed traits at the proximal humerus and even some characters resembling *Lagothrix*, particularly striking in Omo 119–73–2718 [41,77].

When humeral torsion is taken into account (Figs. 7, 8 and Table 6) A.L. 288–1r exhibits a correspondence of degree of humeral torsion (angle) with proximal humeral shape within the range of hylobatids, Sts 7 within the range of *Pongo* but also in the lower end of *H*. *sapiens* values, and Omo 119–73–2718 shows a humeral head shape score close to the higher end of the *Lagothrix* dispersion (Fig. 8), but exhibiting a much higher torsion angle (Fig. 8). Thus, Omo 119–73–2718 exhibits a *Lagothrix*-like humeral shape, but also presents a degree of humeral torsion in the range of *Pongo* and *H*. *sapiens*, again showing a distinctive mix of traits in the humerus as seen in the other two australopith specimens (A.L. 288–1r and Sts 7) and providing further evidence of the mosaic nature of the early hominin postcranial features [14,64–66]. The regression of proximal humeral shape and torsion also provides evidence that the functional features underlying the extensiveness (i.e., mobility) of the humeral head are not related to humeral torsion because an increase of globularity or surface extension of the articular surface is not among the morphological traits that correlate with it [41]. Thus, humeral torsion might be an “orthograde trait” for maintaining a correct orientation of the elbow in upright positions, whereas extensiveness of the articular surface of the proximal humerus for enabling high mobility might be better seen as a suspension-related functional trait.

Of the three australopiths specimens analyzed, only Sts 7 showed some morphological affinities for the proximal humerus with *Pan* in the bgPCA (Figs. 2a, 6), although these have to be viewed with great care because of the great overlap between extant hominoids (Fig. 2a). If further analyses are considered, Sts 7 shows more morphological affinities with *Pongo* and sometimes even with modern humans, than with *Pan* or *Gorilla* (Figs. 3a, 4a, 7 and Table 5). Neither A.L. 288–1r nor Omo 119–73–2718 show morphological affinities with the African great apes (Figs. 3a, 4a and Table 5). This further suggests that hominins could have evolved from a generalized arboreal ancestor rather than a knuckle-walking ancestor, as has been argued [79,80]. Moreover, African great apes exhibit a wide range of locomotor behaviors [81], including all types of below-branch locomotion. Consequently, the morphology of the glenohumeral joint of *Gorilla* and to a lesser extent *Pan* (since this taxon displays greater arboreality [82–84]) mostly reflects the compromise between secondarily acquired terrestriality in a joint primarily adapted to an arboreal lifestyle [39,73,85], a pattern that was not observed in the early hominins included in this study.

Tabun 1 showed virtually the same morphotype as that of modern humans (Figs. 2a, 3a, 4a, 6, 7 and Table 5), exhibiting a medio-laterally longer humeral head, with an increase of surface mostly on the medial aspect [37], which could be related to the lowered neutral position of the arm [14,35,37,70]. Also, the major tubercle is smaller overall, with reduced insertion sites for the rotator cuff muscles, which may indicate an early reduction on the reliance on the active stabilizers of the glenohumeral joint and a decreased importance of the arm abductors (especially the m. supraspinatus, as discussed above [14,33,35,73,86] (Figs. 2a, 8). Such a feature could grant the glenohumeral joint of humans the mobility necessary for engaging in manipulative activities with higher proficiency [74], although it seems that the proximal human humerus is overall less derived than those of, at least, the knuckle-walkers (Figs. 2a and 3a), possibly indicating that the bony morphology of the joint is less strikingly derived than previously thought [14,37].

### The glenoid cavity

The results for the glenoid are more equivocal than those of the humerus, as illustrated by the wide dispersion ranges of the groups in the bgPCA and the Procrustes distances among groups (Fig. 2b and Table 5). The shape of the glenoid does not seem to be driven by locomotor constraints as much as that of the proximal humerus. In all of the analyses (Figs. 2b, 3b, 4b) *Pongo* exhibits morphological similarities of the glenoid cavity with *Lagothrix*, with whom it does not share the same locomotor repertoire. The shape of the glenoid cavity of *Pongo* is certainly narrower and more curved than those of apes, and it exhibits a reminiscence of the lip-like elongation of the cranial aspect (Fig. 2b). However, the distinctive morphology of the glenoid cavity of orangutans could be related to a greater passive stabilization of the joint in abducted postures of the arm, permitting ball-and-socket joint contact in the medial and superior aspect of the proximal humerus [37,70] (Fig. 8). Nevertheless, Robert’s [33] morphocline from slightly piriform to oval-shaped primate glenoids is present in the first axis of the bgPCA (Fig. 2b), but the equivocal overlap between *Lagothrix* and *Pongo*, with the consequent relatively monkey-like morphological affinities of the latter taxon, suggests that caution must be employed when locomotor inferences are attempted based on the glenoid cavity alone (e.g., [33,44]). The fossils show varied affinities for the glenoid cavity, with A.L. 288–1l mostly resembling the great apes (with the exception of *Pongo*; Figs. 2b and 3b and Table 5), Sts 7 resembling *Lagothrix*/*Pongo* and KNM-WT 15000 showing some morphological affinities with hylobatids, particularly the great flatness of the articular surface (Fig. 2b). Nonetheless, when the overall shape of the glenoid is taken into account (Fig. 4b and Table 5), KNM-WT 15000 shows a clear outgroup position (Figs. 3b and 4b), which might indicate that its glenoid morphology is unlike any of the extant taxa. However, when size is taken into account, this hominin appears more similar in glenoid shape to the great apes, even though it is smaller overall (Fig. 5b). Another possibility arises from KNM-WT 15000 being a juvenile specimen; its age was placed at early adolescence at the time of death (see [87] and references therein). The glenoid cavity remains partially cartilaginous until adolescence in humans; therefore, the young age of the Nariokotome child might influence the shape of his glenoid cavity and thus the results because the comparative sample is entirely adult. Studies on the ontogenetic trajectory of the glenoid cavity should be undertaken to assess the growth patterns of this structure to obtain more reliable results. For example, a study by Di Vincenzo and colleagues [88] found that the differences between glenoid morphology between *Homo* species are related to a differential degree of development between the centers of ossification of the glenoid [89] due to an enlarged growth period in modern humans [88], and *Australopithecus* might represent a plesiomorphic condition (in Di Vincenzo and colleagues [88] study represented by *A*. *africanus* and *A*. *sediba* glenoid morphology).

### General considerations

Overall, the australopith specimens analyzed exhibit mosaic traits at the proximal humerus. A.L. 288–1r shows mixed characteristics between the derived condition of humans and a more generalized arboreal pattern, and Sts 7 and Omo 119–73–2718 show mixed arboreal traits, combining some *Pongo*-like features with more generalized characteristics resembling *Lagothrix* (especially in Omo 119–73–2718). The arboreal traits found in the proximal humerus of these three early hominins, however, are mainly related to the sustained use of the arms in overhead positions, which enable the use of a relatively significant amount of below-branch positional behaviors, as argued by some authors (e.g., [4,14,15,22,24,25]). None of the three australopith specimens analyzed shared the morphological condition of the African great apes (*Gorilla* and *Pan*), thus building on the contention that the last common ancestor of hominins and panins could have exhibited a more generalized arboreal locomotor repertoire, instead of knuckle-walking [62–64,90,91].

The shape of the glenoid cavity failed to sort out extant taxa in relation to locomotor categories. Nevertheless, A.L. 288–1l and Sts 7 generally appear more similar to the great apes, and if further evidence from the shoulder girdle elements is considered, their general characteristics appear more similar to the arboreal apes and monkeys than to humans. One of these major features is the cranial orientation of the glenoid facet, which has been repeatedly measured in the Sts 7 specimen, with all studies reporting varied angles ranging from 103° to 125°, indicating that the glenoid of this specimen faced more cranially than in humans [14,92–96]. Not enough of the axillary border was preserved in A.L. 288–1l to measure the orientation of the glenoid, but estimates based on the glenoid orientation with respect to the ventral bar [14,96] suggest that the glenoid would also have been more cranially oriented in this specimen. The same pattern has been found for the juvenile *A*. *afarensis* scapula DIK-1–1 [36,97], as well as in *A*. *sediba* (specimen MH2 [98]). Further arboreal features include a well-developed and laterally placed supraglenoid tubercle, an ape-like angle between the scapular spine and the axillary border, and a clavicle that lacks the characteristic human curvature of the medial end in dorsal view, which indicates that these two australopiths (A.L. 288–1r,l and Sts 7) might have maintained a high shoulder position in a funnel-shaped thorax, in addition to overall ape-like forelimb proportions [14,18,19,98,99]. The evidence presented in this study suggests that the forelimbs of the analyzed australopith specimens (A.L. 288–1r,l, Sts 7 and Omo 119–73–2718) could have been functional when engaging in arm-raising behaviors. In particular, their overall shoulder girdle morphology enabled sustaining abducted positions of the arm without needing to rotate the scapula upwards after the first 90° of arm abduction, as in suspensory apes. The arboreal adaptations displayed throughout the australopith forelimb and thorax have been suggested to pose an advantage for niche exploitation (full adaptation to bipedal terrestriality on the ground, and to suspension/climbing on the trees) in early hominins [100]. In this regard, the relaxation of locomotor constraints on the australopithecine hand proposed by several authors (e.g. [101,102]) does not necessarily preclude the possibility of this genus displaying adaptations to the use of the arms in overhead positions during significant proportions of time. However, further evidence of late Miocene hominins and early *Homo*, as examples of the possible preceding and subsequent morphological conditions, as well as subsequent analyses on kinematics and biomechanics should be included in further studies to test the views conveyed in this study.

## Conclusions

The results of this study show that the early hominins A.L. 288–1, Sts 7 and Omo 119–73–1827 exhibit differential glenohumeral joint morphologies, showing affinities with modern humans, the arboreal apes, and the generalized NWM *Lagothrix*. The morphologies of these early hominins thus display distinctive combinations of primitive and derived characteristics (mosaic morphology) not found in any living great ape taxa. Therefore, the debate about the morphological affinities of early hominins should not be limited to human-like versus African great ape–like morphologies. Instead, morphofunctional studies attempting locomotor inferences on early hominins would benefit from including more generalized primate taxa that might better characterize the evolutionary background of the hominoid lineage. The mosaic nature of the postcranial configurations of hominins might render relatively limited morphofunctional inferences if they are based only on extant great ape genera. Notably, the results of this study extend the contention that hominins could have evolved from an ancestor exhibiting quite generalized arboreal locomotor behaviors instead of the derived repertoire exhibited by the African great apes.

## Supporting Information

S1 Table Procrustes coordinates of all studied specimens for the humerus 3D GM analysis. (TXT)

S2 Table Procrustes coordinates of all studied specimens for the glenoid cavity 3D GM analysis. (TXT)
